# Perturbations in Osteogenic Cell Fate Following Exposure to Constituents Present in Tobacco: A Combinatorial Study

**DOI:** 10.3390/toxics11120998

**Published:** 2023-12-07

**Authors:** Joseph V. Madrid, Madeline K. M. Vera-Colón, Nicole I. zur Nieden

**Affiliations:** Department of Molecular, Cell & Systems Biology and Stem Cell Center, College of Natural and Agricultural Sciences, University of California Riverside, Riverside, CA 92521, USA; joseph.madrid@kansascity.edu (J.V.M.); mvera006@ucr.edu (M.K.M.V.-C.)

**Keywords:** developmental toxicity, osteoblasts, embryonic stem cells, cigarettes, tobacco, smoke solution

## Abstract

Tobacco smoke contains between 7000 and 10,000 constituents, and only an evanescently low number of which have been identified, let alone been evaluated for their toxicity. Recently, the Food and Drug Administration has published a list of 93 chemical tobacco constituents that are harmful or potentially harmful to a number of cellular processes. However, their effect on developing skeletal cells is unknown. In this study, we used ToxPI, a computational tool, to prioritize constituents on this list for screening in osteogenically differentiating human embryonic stem cells and fibroblasts. In selected endpoint assays, we evaluated the potential of these chemicals to inhibit osteogenic differentiation success as well as their cytotoxicity. Six of these chemicals, which were ascribed an embryotoxic potential in our screen, as well as nicotine, which was not found to be osteotoxic in vitro, were then evaluated in combinatorial exposures, either in pairs of two or three. No one single chemical could be pinpointed as the culprit of reduced calcification in response to tobacco exposure. Combining chemicals at their half-maximal inhibitory concentration of differentiation often elicited expected decreases in calcification over the individual exposures; however, cytotoxicity was improved in many of the dual combinations. A reverse response was also noted, in which calcification output improved in combinatorial exposures. Results from ternary combinations reflected those from double combinations. Thus, the results from this study suggest that it may be difficult to isolate single chemicals as the primary drivers of skeletal embryotoxicity and that the full combination of chemicals in tobacco smoke may produce the hypomineralization phenotype that we have so far observed in vitro in human embryonic stem cells as well as in vivo in zebrafish.

## 1. Introduction

Tobacco smoke exposure is responsible for 480,000 deaths annually and is reported by the US Surgeon General as the most extensively studied environmental source of disease [1,2,3,4,5]. The diseases implicated or exacerbated by tobacco use are ample, such as heart disease, stroke, multiple organ-specific cancers, premature aging, and infertility [5,6]. Smoking may also disrupt osteogenesis and can lead to osteoporosis, an increase in bone fragility, and an overall delay in bone healing [7,8].

Despite these known health consequences of tobacco use, 10–20% of pregnant women report smoking during their first trimester of pregnancy [9]. This is concerning since in utero and early-in-life exposure to tobacco are linked to still birth and sudden infant death syndrome [10]. Aside from mortality, numerous additional adverse pregnancy outcomes are associated with in utero tobacco or second-hand exposure, including early delivery, low birth weight, and congenital heart defects [11,12].

More recent reports also relate in utero tobacco exposure to skeletal manifestations in newborns, including a decrease in overall body height and head and chest circumferences [13]. Bones are often hypomineralized, as evidenced by lower bone mineral density and bone mineral content, for example, in the head; consequently, the risk of fracture following minor injuries is increased [14,15,16,17]. We have recently been able to model these human epidemiological phenotypes in developing zebrafish, which exhibited hypomineralization in multiple bones, alterations to cartilage architecture, and spinal deformations in response to tobacco exposure [18].

The last few decades have seen multiple efforts to reduce the adverse health outcomes associated with tobacco consumption, first and foremost to decrease the amount of harmful chemical constituents. For instance, harm-reduction cigarettes are manufactured to contain less tar and nicotine [19]. On 22 October 2019, the Food and Drug Administration (FDA), for the first time, approved some Snus products—smokeless tobacco pouches that are placed under the lip to allow absorption of nicotine—to be marketed as bearing reduced harm in comparison to combustible cigarettes [20]. This designation is in part based on the fact that smokeless tobacco contains fewer constituents and lower concentrations of chemicals compared to conventional tobacco products. Its product packaging also specifies a lower risk of mouth cancer, heart disease, lung cancer, stroke, emphysema, and chronic bronchitis. However, exposure to Snus or other harm-reduction products is not completely without risk; therefore, there is an urgent need to identify additional harmful constituents that could be omitted during manufacture of any tobacco product.

Historically, human exposure risk to hazardous chemical compounds has been experimentally assessed in rodents. However, since the mid-20th century there has been a push to reduce the amount of animal use and suffering for scientific purposes. The concept of the Three Rs (Replacement, Reduction, and Refinement) outlines the importance of refraining from animal use and, when possible, use in vitro and prediction models to perform experiments [21,22]. Approaches to embryotoxicity screening include the embryonic stem cell test (EST) [23,24], which completely avoids the use of animals during testing. To classify compounds as embryotoxic to the developing skeleton, the EST uses three endpoints: (1) viability of chemical-treated adult fibroblasts, (2) viability of chemical-treated ESCs, and (3) differentiation capability of chemical-treated ESCs [25,26,27,28]. Dose–response curves are obtained for half-maximal cytotoxicity (IC_50_) and differentiation inhibition (ID_50_). Using an EST based on a human embryonic stem cell (hESC) osteogenic differentiation model (hESTo), we have recently screened smoke extracts from three conventional and two harm-reduction cigarettes and found associated osteogenic differentiation inhibition and hypomineralization in all five products [29], a phenotype akin to that observed in epidemiological studies in humans. Innovative video bioinformatic analysis further identified N’-nitrosonornicotine as a potential chemical contributing to the skeletal embryotoxicity of the tobacco extracts [30]. This human ESC differentiation model thus seems ideally suited to establish the skeletal toxicity associated with other chemical constituents found in tobacco.

However, overall, it is challenging to decipher how an individual chemical in mainstream tobacco or environmental smoke contributes to toxicity or teratogenic effects during embryogenesis. A major obstacle in studying these effects is the tobacco smoke itself, a concoction composed of more than 7000 chemical constituents. With the passage of the Family Smoking and Prevention Act, the Food and Drug Administration (FDA) released a list of harmful and potentially harmful constituents (HPHCs) in tobacco products and tobacco smoke in 2009. While most of the chemical constituents in tobacco smoke and products remain incompletely identified, the HPHCs may provide a good framework for focusing on those chemicals that pose the greatest threat to human health and might represent an excellent starting point for assessing embryotoxicity risk associated with exposure to individual chemical constituents found in tobacco. Starting with the HPHC list, we attempt here to experimentally identify single constituents found in tobacco that inhibit skeletogenesis in vitro.

While the advantage of using hESCs to gauge skeletal toxicity associated with chemical exposure over other in vitro alternatives is apparent in the relevance to human-specific exposure, the relatively long culture duration qualifies this model as medium-throughput. Therefore, testing all chemical constituents found in tobacco, even if identified, would be a lengthy and costly undertaking. The identification of potentially harmful tobacco constituents in the HPHC list has substantially reduced the number of chemicals to test down to 93, yet testing these chemical constituents individually or in assortments would still be time-consuming and expensive. Therefore, to prioritize chemicals for toxicity testing, we applied here the Toxicology Priority Index (ToxPI) graphical user interface developed by the Environmental Protection Agency (EPA) in conjunction with the University of South Carolina at Chapel Hill [31] using data from Tox21 and Toxcast and then screened the prioritized hits within the human in vitro skeletal toxicity model.

## 2. Methods

### 2.1. Cell Passaging and Maintenance

Human embryonic stem cells of the line H9 (WiCell) were seeded on Matrigel-coated culture plates and cultured in mTeSR1 medium (Stem Cell Technologies, Seattle, WA, USA) at 37 °C with 5% CO_2_. Approximately every four days, colonies were treated with accutase for 2–4 min at room temperature to dislodge colonies from the plastic and passaged into fresh Matrigel-coated wells. Human foreskin fibroblasts (hFF-1, American Type Culture Collection, Manassas, VA, USA, cat.no. SCRC-1041), were cultured on 0.1% gelatin-coated 6-well plates in Dulbecco’s Modified Eagle Medium (DMEM) with 10% fetal bovine serum, 1% (*v*/*v*) non-essential amino acids, 50 U/mL penicillin, and 50 µg/mL streptomycin at 37 °C in a controlled humidified 5% CO_2_ incubator. Upon 70% confluency, hFFs were passaged at a 1:6 ratio using phosphate-buffered saline (PBS) washes and 2.5% Trypsin/EDTA every two to four days. While in culture, all cells were routinely checked for bacterial, yeast, and microbial contamination using an inverted microscope and tested for mycoplasma using a PCR-based detection kit (abcam, Fremont, CA, USA, ab289834).

### 2.2. Osteogenic Differentiation of Human Embryonic Stem Cells

Differentiation was induced from hESC cultures through the addition of a control differentiation medium (CDM) composed of DMEM, 15% FBS (Atlanta Biologicals, Flowery Branch, GA, USA), 1% (*v*/*v*) non-essential amino acids, 50 U/mL penicillin, 50 µg/mL streptomycin, and 0.1 mM β-mercaptoethanol. Five days after this initial medium change, CDM was supplemented with 1.2 × 10^−7^ M 1,25α(OH)_2_ vitamin D_3_ (Calbiochem, Burlington, MA, USA), 0.1 mM β-glycerophosphate, and 20.8 µg/mL ascorbic acid [32].

### 2.3. Chemical Exposure

Cells were cultured with concomitant chemical treatment in a double-blind, vehicle-controlled study. Acetaldehyde (Sigma Aldrich, St. Louis, MO, USA, cat. no. 402788), acrolein (Sigma Aldrich, cat. no. 190543), acrylamide (Sigma Aldrich, cat. no. 23701), all-trans retinoic acid (Sigma Aldrich, cat.no. R2625), benz[a]anthracene (Santa Cruz Biotechnology, Inc., Dallas, TX, USA, cat. no. sc-252409), benzo[a]pyrene (Toronto Research Chemicals, Manchester, NH, USA, cat. no. B205800), benzo[b]fluoranthene (Toronto Research Chemicals, cat. no. B209865), catechol (Santa Cruz Biotechnology, cat. no. sc-215763), cotinine (Sigma Aldrich, cat. no. C5923), coumarin (Santa Cruz Biotechnology, cat. no. sc-205637), isonicotine (Sigma Aldrich, cat. no. I17508), nicotine (Toronto Research Chemicals, cat. no. sc-N0267), penicillin G sodium salt (Sigma Aldrich, cat. No. P3032), quinoline (Sigma Aldrich, cat. no. 241571), and urethane (Sigma Aldrich, cat. no. U2500) were purchased from the indicated companies. Stock solutions were made in DMEM or dimethylsulfoxide, depending on chemical solubility, sonicated when necessary, sterile filtered (0.1 µm), and stored at −20 °C. For liquids, concentrations were calculated from their density. Final dilutions were made in appropriate cell culture medium upon additional sonication of the stock after thawing. Chemical cell treatment started on day 0 of culture/differentiation and continued through day 20 in both H9 hESCs and hFF cells. Medium, including chemical dilutions, were made fresh and changed every other day. All exposures were performed in biological triplicate, each containing technical quintuplicates.

### 2.4. Detection of Calcium

Quantification of calcium in the extracellular matrix of the cultures was performed using a calcium assay normalized to protein content. First, cells were lysed with radio-immunoprecipitation buffer (1% NP40, 0.5% sodium deoxycholate, 0.1% sodium dodecyl sulfate in PBS) on day 20 of cellular differentiation. The remaining matrix was dissolved with 1 N HCl and collected. Both lysates were assayed with Arsenazo III (Genzyme), and absorbance was measured at 655 nm (iMark microplate reader; Bio-Rad Laboratories, Hercules, CA, USA). Absorbances were compared to a CaCl_2_ standard, and total calcium content was normalized to the total protein content of the sample determined by a Lowry assay, measured against a bovine serum albumin standard curve [33]. The Lowry assay was read at 750 nm (iMark microplate reader; Bio-Rad Laboratories) after a 15 min micro-shake incubation. Calculation of final normalized calcium content was performed according to a previously published formula [33].

### 2.5. MTT Assay

Viability response to constituent exposure was determined by 3-[4,5-dimethylthiazol-2yl]-2,5-diphenylterazolium bromide (MTT) assay. Cells were incubated with MTT (5 mg/mL) at 37 °C for 2 h. Following incubation, the supernatant was removed and replaced with 0.04 mol/L HCl in isopropanol. The plate was placed on a shaker for 15 min to dissolve aggregates. The optical density of the solution was read at 595 nm (iMark microplate reader; BioRad) [26,27,28,34,35].

### 2.6. Toxicity Forecaster Data Mining

The Environmental Protection Agency’s Aggregated Computational Toxicology online resource (ACToR) was accessed at https://actor.epa.gov/actor/home.xhtml on 20 September 2017. The Toxicity Forecaster (ToxCast) dashboard link was selected. Data for the ToxPI software were mined using the Aggregated Computational Toxicology Online Resource (ACToR) database (https://actor.epa.gov/actor/home.xhtml, accessed on 20 September 2017) and were exported using the interactive Chemical Safety and Sustainability (iCCS) table, which contains data from multiple database sources including Toxicity Forecaster (Toxcast) and Toxicity Testing in the 21st century (Tox21).

Information in the iCCS database is given in activity call (AC_50_) values, which are indicative of either inhibitory or activation effects. Selected chemicals were individually inserted into the chemical name search bar. Chemicals were selected with the advanced search bar, which prompted an additional window to appear. Under “Filter assay”, using biological processes target was selected from the drop-down menu. From the on-value section, five biological processes were selected from the drop-down menu: cell death, cell proliferation, cell morphology, oxidative phosphorylation, and mitochondrial depolarization. Information for selected chemicals was viewable under the chemical and assays tabs and downloaded using the Export tab at the top right corner of the browser. For each chemical the entire process was repeated with a new browser window. All downloaded data were reformatted into a single Excel .csv file for compatibility with the ToxPI GUI.

### 2.7. ToxPI Pie Chart Generation

**Figure 1:**
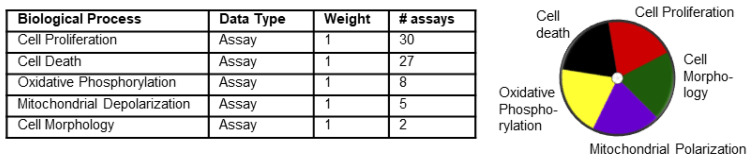
Five assays used for generation of ToxPI charts. The table lists the parameters loaded into ToxPI. Each of the five selected biological processes were given their own pie chart slice based off assay data and had equal weight. ToxPI charts were generated for 17 chemicals using 72 in vitro assays across 5 biological processes. The image on the right represents the color code used for each of the processes. Each slice was given its own color.

The ToxPI GUI was downloaded at http://comptox.unc.edu/toxpi.php (accessed on 20 September 2017) and saved as a Java application on the desktop. Data exported from the Toxicity Forecaster were reformatted in an Excel sheet and inserted into the ToxPI program. The ToxPI GUI was opened, and the reformatted Excel sheet was uploaded by the “insert” icon. In total, seventeen chemicals were chosen manually, and the “generate charts” icon was selected, which allowed each of the biological processes to be sorted. The data were sorted by the assays option, and each assay option was named for each biological process. For each assay option, a corresponding data selection icon appeared, and data for each biological process were selected manually. Each biological process was given a uniquely colored pie slice (Figure 1): cell morphology, 2 assays; mitochondrial depolarization, 5 assays; oxidative phosphorylation, 8 assays; cell death, 27 assays; and cell proliferation, 30 assays. These 5 pie slices were scaled by selecting −log10(x) + log10(max(x)) on the drop-down menu; this ensured that lower active AC_50_ values were given separation from non-active hits. Pie charts were generated by selecting the final generation tab.

### 2.8. Statistical Analysis

Half-maximal inhibitory doses of cytotoxicity (IC_50_) and osteogenic differentiation (ID_50_) were taken from concentration–response curves generated in GraphPad Prism (9.2., GraphPad Software Inc., San Diego, CA, USA) with the “nonlinear regression” function). A biostatistical prediction model based on linear discriminant functions was employed to classify the test chemical into one out of three embryotoxicity categories (strong, weak, and non-embryotoxic) [24]. In double combination analyses, a *t*-test was performed comparing combination to single constituent ID_50_. For triple combination analyses, a one-way ANOVA with Dunnett’s multiple comparison test (single constituent vs. combination) using GraphPad Prism was conducted. A *p*-value below 0.05 was considered significant. Heatmaps for double and triple exposures were generated in GraphPad Prism 9.2. Calcium-to-MTT ratios were calculated in Microsoft Excel and imported to ClustVis [36] to generate a principal-component-analysis-based clustered heatmap.

## 3. Results

### 3.1. Dose–Response Curves Revealed Skeletal Embryotoxicity for ToxPI Positive Chemicals

**Figure 2:**
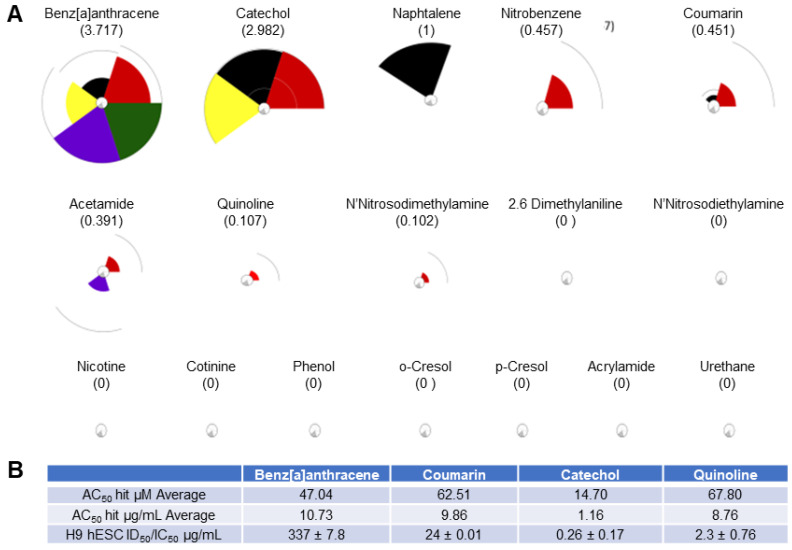
ToxPI results for 17 HPHCs. (**A**) Pie charts for 17 of the chemicals on the HPHC list. Refer to Figure 1 for color coding of ToxPI pie slices. (**B**) ToxPI charts. Benz[a]anthracene (3.717) through N-nitrosodimethylamine (0.102) had designated “hits” and produced positive ToxPi values (0 < X). The remainder of the chemicals, including nicotine, had ToxPI-predicted null effects (X = 0). (**B**) Toxicity Forecast data averages and in vitro H9 hESC ID_50_/IC_50_ µg/mL values obtained from concentration–response curves. The average assay hit value in µM (AC_50_) for each of the tested ToxPI positive constituents was obtained from the Toxicity Forecaster database. The AC_50_ in µM for was then converted into µg/mL for appropriate cross-comparison to the H9 hESC ID_50_/IC_50_ values calculated from the in vitro osteogenic screen (see page 8).

Of the 93 chemicals on the FDA’s HPHC list, only 46 were found in the Toxicity Forecaster database; however, only 17 of those had complete data sets (Figure 2). For each of these chemicals, 72 assays were exported from the Toxicity Forecaster dashboard across the five biological processes. Figure 2A shows their ranking according to most likely to be harmful on the left (multiple large pie pieces) to least likely to be harmful on the right (no pie pieces). Benz[a]anthracene (3.717) through N-nitrosodimethylamine (0.102) had designated “hits” and produced positive ToxPI values (0 < X). The remainder of the chemicals were identified with null effects (0 = X).

To then validate the ToxPI predictions, the hESTo was used. Initial experiments were undertaken with a negative and positive reference chemical to ensure the adequate response of the human cells. Penicillin G (non-embryotoxic) and all-trans retinoic acid (positive reference chemical) have been used as reference chemicals before, with all-trans retinoic acid showing concentration-dependent toxicity on the osteogenic lineage [23,24,25,26,27,28].

First, hESCs were induced to undergo osteogenesis from overgrowing cultures with the addition of vitamin D3. This differentiation protocol, while primarily generating osteoblasts from the neural crest [31], will still form osteoblasts derived from the mesoderm and will therefore identify chemicals with the potential to disturb any type of skeletal formation.

**Figure 3:**
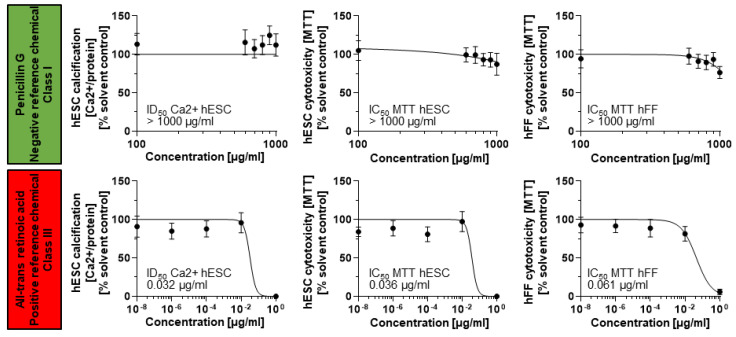
Differentiation and cell viability endpoints measured for a negative and positive reference chemical. Mitochondrial dehydrogenase activity and calcium content (mg Ca^2+^/mg protein) in response to chemical exposure charted as average ± SD from *n* = 15 technical replicates representing three biological replicates. hESC, human embryonic stem cell; hFF, human foreskin fibroblast; MTT, 3-[4,5-dimethylthiazol-2-yl]-2,5-diphenylterazolium bromide.

Half-maximal inhibitory concentrations for cell viability (IC_50_ hESC) and differentiation (ID_50_ Ca^2+^ hESC) were established for penicillin G and all-trans retinoic acid from concentration–response curves. The hESTo then compares these two endpoints to the half-maximal inhibitory concentration for cell viability resulting from exposure of fibroblast cells to the same test compound (IC_50_ MTT hFF) (Figure 3).

Penicillin G exposures did not result in the identification of corresponding IC and ID_50_ values in any cell type. Therefore, its half-maximal inhibitory concentrations were set to the maximally achieved exposure concentration of 1000 µg/mL. The three endpoints were then summarized in a biostatistical model that allows for the classification of the test compound into three embryotoxicity classes: non-, weak, and strong embryotoxic [24]. This biostatistical model classified the two reference chemicals correctly according to their in vivo embryotoxic potential: non-embryotoxic for penicillin G (class I) and strongly embryotoxic for all-trans retinoic acid (class III), suggesting that the hESTo may serve as an appropriate predictor for human exposure risk.

Next, benz[a]anthracence, catechol, coumarin, and quinoline were randomly selected for further analysis from the seven ToxPI positive chemicals. The MTT and calcium assay, corresponding to cell viability and osteogenic differentiation, respectively, revealed that three of the four ToxPI positive chemicals (0 < X) exhibited severe concentration-dependent responses in cytotoxicity and differentiation inhibition. While H9 hESCs showed a decrease in calcification and cytotoxicity in response to benz[a]anthracene as with the other three chemicals, the decrease occurred at a relatively high concentrations—the IC_50_ and ID_50_ were determined at 337.3 µg/mL and 239.5 µg/mL, respectively.

**Figure 4:**
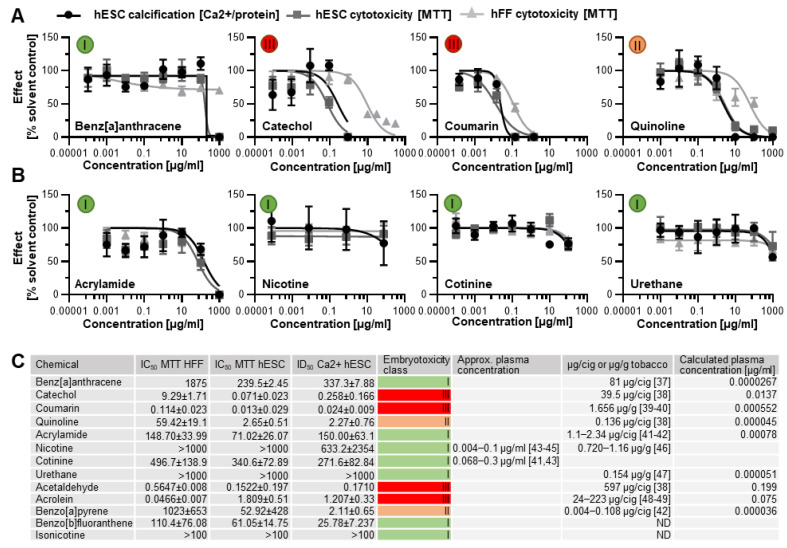
Concentration–response curves. (**A**) Three ToxPI positive constituents elicited an effect on cytotoxicity and osteogenic differentiation. (**B**) All of the ToxPI null chemicals caused inhibition at relatively high concentrations. (**C**) Table listing IC_50_ and ID_50_ values, calculated via GraphPad Prism and expressed as µg/mL, embryotoxicity classes calculated according to Genschow et al. [24], and content per cigarette (cig) or gram tobacco as well as actual and calculated plasma concentrations assuming a plasma volume of 3000 mL. Embryotoxicity classes are given in colored circles. ID_50_ = half-maximal inhibitory concentration of differentiation. IC_50_ = half-maximal concentration of cytotoxicity. hESC, human embryonic stem cell; hFF, human foreskin fibroblast; ND, not determined from the literature [37,38,39,40,41,42,43,44,45,46,47,48,49].

With the concentration–response curves of H9 cell viability and calcification following similar trends, it is also likely that the decrease in calcification may be an attributed consequence of the overall reduction in viable cells. Against ToxPI predictions, benz[a]anthracene exposure did not produce a change in cell viability toward the hFF cell line for the tested concentration range up to 1000 µg/mL (Figure 4A). It is often the non-embryotoxic chemicals that produce half-maximal inhibitory concentrations in such high concentration ranges [25,28,34], and indeed a biostatistical model classified benz[a]anthracene as non-embryotoxic to skeletal differentiation.

Catechol exposure caused a decrease in calcification and viability that went in line with the ToxPI predictions. Catechol produced a change in cell viability toward the hFF cell line above 0.882 µg/mL (Figure 4A). Around this concentration range, cytotoxicity and differentiation inhibition with the hESC cell line were already below 25% of the solvent control. The dose–response curves for hESC viability and calcification closely followed each other; hence, any developmental osteotoxicity may be caused by the general cytotoxicity of the compound. The biostatistical model predicted catechol as strongly embryotoxic (Figure 4C).

For coumarin, the ToxPI program predicted marginal toxicity; thus, expectations for the experimental validation of its toxicity were uncertain. Experimentally, we found that hFF and H9 cell viability and H9 differentiation potential were reduced in the same concentration range, with the hFFs being slightly less sensitive to the compound. With the dose–response curves of H9 cell viability and calcification again following similar trends, it is believed that the decrease in calcification might be an attributed consequence of the overall reduction in viable cells. Thus, a 0.013 µg/mL IC_50_ was determined for the H9 hESC line. hFF viability was also reduced as the concentration of coumarin increased. Linear interpolation determined an IC_50_ of 0.114 µg/mL for hFF cells. To ultimately judge the danger associated with coumarin exposure to the developing skeleton, again, the biostatistical model was applied. As in the case of catechol, it generated an embryotoxicity class of III, strong embryotoxic.

Quinoline also elicited greater sensitivity in the hESC endpoints than in the hFF cells, and again the curve for the differentiation inhibition closely followed the cytotoxicity curve. Yet, the half-maximal inhibitory concentrations ranged about a decade higher than for coumarin and catechol. Interestingly, the predictive value of each ToxPI assay hit rate in µg/mL (Figure 1C) was relatively close to the half-maximal inhibitory concentrations experimentally observed in vitro for the H9 hESCs for all four tested chemicals.

### 3.2. Human In Vitro Data for ToxPI Negative Chemicals

Four ToxPI null chemicals (nicotine, cotinine, urethane, and acrylamide) were also taken through in vitro screening. ToxPI-predicted null effects matched in vitro screen data, as urethane, cotinine, and nicotine exposure did not inhibit cell viability for either the H9 or hFF cell lines for all tested concentration ranges (Figure 4B). Likewise, H9 hESC differentiation potential was not altered in the same concentration range for urethane and cotinine. However, a decrease in calcification was seen in the higher concentrations of nicotine exposure. Acrylamide, in turn, caused reductions in cell viability as well as calcification but in very high concentration ranges. Of these four chemicals tested, all were predicted to have non-embryotoxic effects when employing the biostatistical model (Figure 4C), in line with the ToxPI predictions.

### 3.3. Screening of Additional Tobacco Constituents

**Figure 5:**
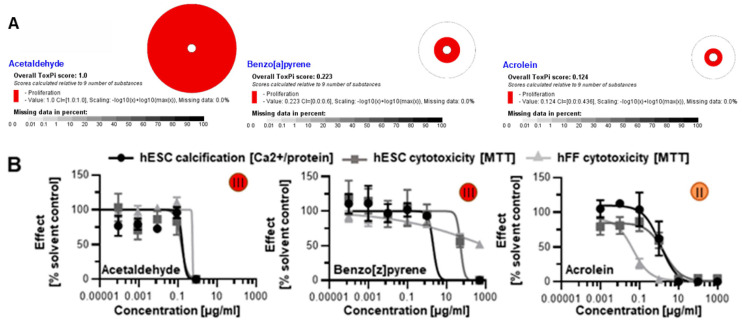
Skeletal embryotoxicity predictions for additional tobacco smoke constituents with existing ToxCast data. (**A**) All three constituents were forecasted to elicit embryotoxicity based on ToxPI predictions. (**B**) Screening with the hESTo classified all three constituents as developmentally osteotoxic. All curves were graphed via GraphPad Prism. Embryotoxicity classes [24] are given in colored circles. hESC, human embryonic stem cell; hFF, human foreskin fibroblast.

In addition to the 17 chemicals we identified from the HPHC list using ToxPI, we also selected five more based on existing developmental and skeletal toxicity data: acetaldehyde [50,51], benzo[a]pyrene [52,53], acrolein, benzo[b]fluoranthene [54], and isonicotine. The first three were existent in the database, though with incomplete assays. Using AC_50_ concentrations from the available proliferation assays generated positive ToxPI charts (Figure 5A).

**Figure 6:**
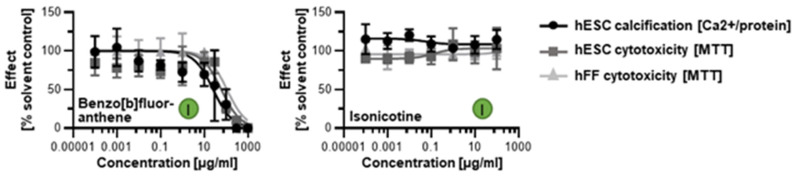
hESTo data for additional tobacco smoke constituents with non-existing ToxCast data. Curves were graphed via GraphPad Prism. Embryotoxicity classes [24] are given in colored circles. hESC, human embryonic stem cell; hFF, human foreskin fibroblast.

When screened with the human in vitro model of skeletogenesis, acetaldehyde, benzo[a]pyrene, and acrolein caused differentiation inhibition and cytotoxicity as predicted (Figure 5B) Notably, differentiation was inhibited at lower concentrations than viability in the case of benzo[a]pyrene exposure, potentially pointing to a differentiation defect that is decoupled from cell death. In turn, benzo[b]fluoranthene and isonicotine were not found in the database and, therefore, ToxPI charts could not be generated. Upon testing in the hESTo, neither chemical classified as developmentally osteotoxic (Figure 6). 

### 3.4. Double Combinatorial Exposures Elicit Apparent Synergistic or Antagonistic Effects

It remains to be seen whether it really is only a single chemical constituent in tobacco smoke that causes the calcification defects or whether it is a mixture of harmful chemicals, whereby some exert positive and others negative effects. To begin answering this question, we next tested combinations of chemicals starting with the chemicals identified as carrying a skeletal embryotoxicity risk from Figure 4C. Nicotine was also included due to its prevalence in tobacco products and its highly addictive properties, despite its classification as a ToxPI null chemical.

**Figure 7:**
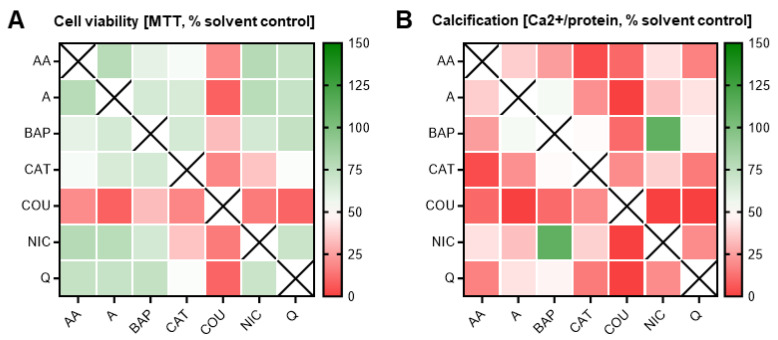
Double combination heatmaps. MTT (**A**) and calcium (**B**) assays corresponding to double combinatorial exposures. In most combinations, cell survival was maintained at or above 100%; however, differentiation was inhibited in almost all combinations. Heatmaps were generated via GraphPad Prism. Data were normalized to solvent control (0.1% DMSO). AA, acetaldehyde; BAP, benzo[a]pyrene; CAT, catechol; COU, coumarin; NIC, nicotine; Q, quinoline; A, acrolein.

Dosed at obtained ID_50_ concentrations, 21 different double combinations were tested. As predicted, cytotoxic and differentiation inhibitory effects were seen when combining two chemicals. However, most notable observations depict an overall maintenance of cell survival between the expected 50–75% mark amongst many of the combinations (Figure 7), except those that contained coumarin. 

Cells exposed to any coumarin dual combination, consequently, were also characterized by a calcification output lower than the expected 50%. Of note, in many of the other chemical pairs, cell survival was maintained at levels not statistically different from the individual exposures (i.e., 50%). For instance, despite the individual constituents, quinoline and catechol both identified as ToxPI positive components and elicited an ID_50_ at a relatively low concentration (indicating high toxicity), and the combinatorial pair had no additive effect on cell survival (Figure 6). 

However, there was a notable decrease in osteogenic differentiation efficiency. In fact, catechol was identified as a positive ToxPI constituent, and in most pairs with catechol, differentiation was significantly further reduced beyond 50%, at non-lethal concentrations. One of the few exceptions from this pattern was exposure to catechol in a pair with nicotine, an identified ToxPI null chemical, which resulted in a significant decrease in differentiation as well as viability. This may suggest that two constituents that do only mildly affect cell viability by themselves may elicit toxicity when combined.

Lastly, benzo[a]pyrene, a well-known carcinogen, only significantly reduced differentiation below 50% in combination with three of the chemicals (acetaldehyde, catechol, and coumarin) and in only one instance (coumarin) reduced cell viability, potentially pointing to antagonistic properties of this constituent. Further underlining this possibility is the fact that benzo[a]pyrene, in combination with nicotine, rescued calcification to solvent control levels. Thus, overall, the double combination exposures highlighted key combinations (and select chemicals) that demonstrated worsened effects to skeletal embryotoxicity, while a few also counteracted each other to cancel out skeletal embryotoxicity.

### 3.5. Addition of a Third Constituent Elicited Different Effects from Two-Constituent Combinations

**Figure 8:**
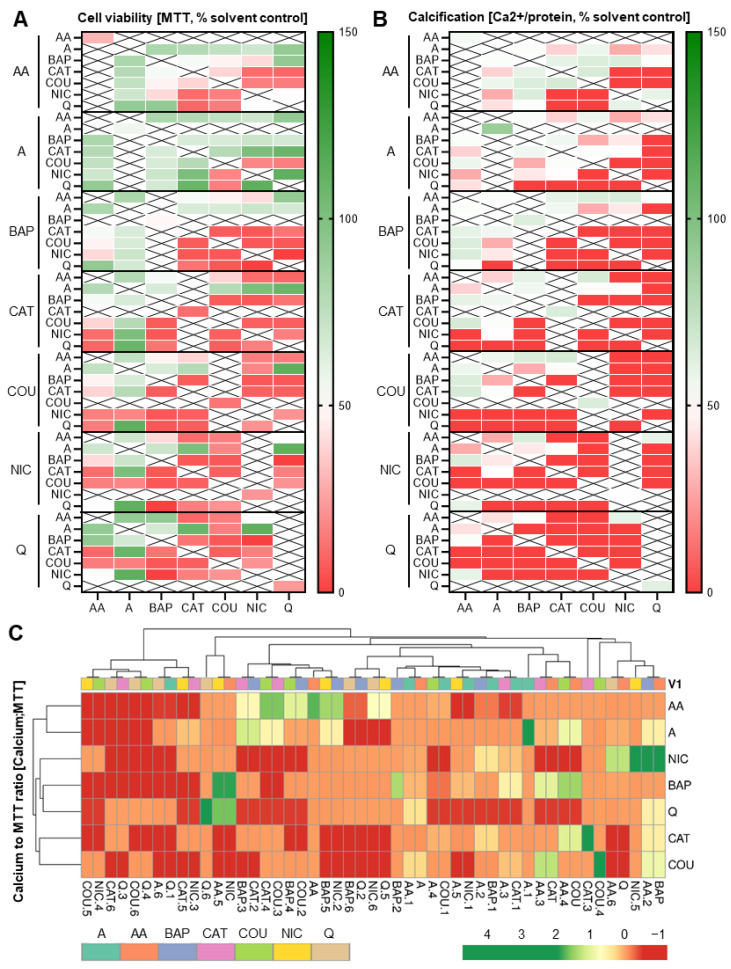
Heatmaps pertaining to triple exposure combinations. (**A**,**B**) Triple combination heatmaps. MTT (**A**) and calcium (**B**) assays corresponding to triple combinatorial exposures. Heatmaps were generated via GraphPad Prism. In most combinations, the addition of acrolein (in comparison to double combinations) maintained cell survival at or above 100%. Data were normalized to solvent control (0.1% DMSO). (**C**) Calcium content was set in relation to MTT results and a clustered heatmap was generated from the resulting ratios using ClustVis [36]. AA, acetaldehyde; BAP, benzo[a]pyrene; CAT, catechol; COU, coumarin; NIC, nicotine; Q, quinoline; A, acrolein.

Since it is likely that additional co-exposures further change the overall embryotoxic outcome to differentiating skeletal cells, a series of ternary combinations was explored next. Dosed at individual ID_50_ concentrations, seven different sets of triple combinations were tested. Cytotoxicity and differentiation inhibition further decreased with the addition of a third chemical with respect to most of the double combination exposures (Figure 8A). Again, differentiation inhibition was often found in the absence of compounded cytotoxic effects. For instance, above, we highlighted catechol as affecting differentiation and not viability; however, in most triple combination pairs, most notably with quinoline and nicotine, exposures also resulted in cell viability responses below 50%. In contrast, the triple combination pair containing catechol and acrolein remained mostly unchanged or was even “rescued” despite differences in the third chemical. The differentiation endpoint followed suit with the same observed patterns in these combinations.

Most ternary combinations with coumarin potently affected viability and differentiation, as observed for the double combinations. However, when paired with acetaldehyde, independently of the third chemical, this trend was reversed. Interestingly, both acrolein and acetaldehyde seemed to have an overall positive influence on cell survival and differentiation outcome when added to most double combinations. In contrast, nicotine elicited mostly an overall decrease in viability and thereby differentiation in most of its ternary combinations.

To determine which of the chemicals caused a differentiation defect without a decrease in viability, we next expressed the two endpoints as ratios of each other and created a clustered heatmap (Figure 8B). This clustered heatmap illustrated that most of the chemical combinations generated a differentiation defect while relatively preserving cellular viability. However, in turn, it also identified chemicals that decreased cellular viability while seemingly preserving calcification output. The most prevalent of such examples were the acetaldehyde combinations, often together with benzo[a]pyrene. Together, these ternary combinatorial data prove the unpredictable nature of skeletal embryotoxicity when hundreds or thousands of constituents are present, as is the case for tobacco smoke.

## 4. Discussion

Narrowing down the primary drivers of the chemical mixture that encompasses tobacco products and smoke remains a large challenge. This study has found that using the ToxPI program in conjunction with Toxcast data can partition cytotoxic constituents from those that will produce no harm to osteogenically differentiating cells within their predictive range. Though the parameters chosen to generate the ToxPI charts were not specific to the osteogenic lineage, their testing in a well-established protocol was a recapitulation of osteogenic development. With the assimilation of Toxcast data in their infancy, the database has the potential to grow and could have data available in the future to help predict osteogenic maldevelopment specifically as seen currently with other target tissues.

Nonetheless, our results suggest the importance of actual screening is not to be underestimated, given that 1 out of the 11 chemicals that we tested in vitro classified incorrectly with either assay. This chemical was benz[a]anthracene. That this chemical classified incorrectly is concerning given that B(a)A is considered a high-priority chemical by the European Union and US Environmental Protection Agency [55]. Long known as a mutagen and for its genotoxicity [56,57], more recent reports have documented developmental defects upon exposure [55]. Specifically, medaka larvae were found to incur spinal and craniofacial malformations, pointing to an issue with skeletal development. Given these in vivo results, the in vitro screening results are highly likely to have provided a false negative outcome. In the EST, false negatives are typically associated with chemicals that need to be biotransformed to become toxic [58], as most metabolic activation enzymes are absent from this cell system [59]. Indeed, B(a)A undergoes biotransformation in aquatic species [60] to more toxic metabolites [61,62,63]. Since B(a)A is thus classified as a pro-teratogen, it could be possible that B(a)A exposure in hESCs would result in a category III classification if cultivation would occur with the addition of an S9 mix made from microsomal and cytosolic fractions of liver cells [64] that could metabolize it properly.

While a 100% overlap between the ToxPI predictions and the hESTo results under current conditions was not achieved, the ToxPI predicted AC_50_ and in vitro H9 hESC IC_50_ values did correspond well with numerous in vivo tests. Coumarin’s toxicity in zebrafish and Xenopus provided half-maximal activity values for viability that were adjacent to both our own hESC and hFF IC_50_ values [65,66]. Likewise, quinoline is reported to induce a variety of malformations and toxicity between 29 and 95 µg/mL depending on the developmental stage of Xenopus [67], where the median lethal dose for other aquatic species has been reported to be 11 µg/mL [68]. Catechol had a variety of animal models [69,70] with toxic concentrations similar to our own, and it corroborated with other independent in vitro data [71]. B(a)A also had exposure values that resulted in the death of zebrafish larvae slightly lower than our results [72].

The actual plasma concentrations reached after exposure to the two constituents identified as weakly developmentally osteotoxic (quinoline and benzo[a]pyrene) might not be sufficient to elicit developmental defects in vivo. At concentrations of 0.136 and 0.108 µg/cigarette, respectively (Figure 2C) [38,42], and an assumed average human plasma volume of 3000 mL, exposure to a dose of 1 g would cause plasma concentrations 50,000- and 580,000-fold less than our identified ID_50_ values. In the case of the four constituents identified as strongly developmentally osteotoxic (catechol, coumarin, acetaldehyde, and acrolein) however, calculated plasma concentrations after exposure are either on par with the identified ID_50_ concentrations (acetaldehyde) or only 16–45-fold removed from them. Indeed, the results for these chemicals were in line with their known developmental effects or were able to clarify their toxic potential further. 

For example, catechol is a natural phenolic compound found in trace amounts in fruits and vegetables, where it is bacterially synthesized from glucose or broken down from catechins [73,74,75]. It can also be synthetically manufactured from fossil fuels through bacterial transformation for uses from perfumes to pesticides [76]. As a major metabolite of phenol, catechol induces dysmorphogenesis in cultured rat embryos and half-maximal lethality in the range of 50–100 µM [77]. Additional reports on the developmental toxicity of catechols or their target tissue specificity seem scarce. Instead, catechols are being investigated for their beneficial antioxidant and anti-glycemic activity [78,79,80,81,82]. However, catechin-rich extracts of turmeric, whose rhizomes are used in cooking and some medicinal applications, produce kinked tails and bent trunks in zebrafish [83]. It remains to be understood whether these defects are produced via a different chemical mechanism or whether it is the excessive oxidant removal and glucose-lowering action that affects development negatively. Indeed, we have seen this in control conditions run alongside our own experiments: osteogenic hESC cultures dosed with various antioxidants all exhibited hypomineralization [84], potentially suggesting that a healthy level of oxidants is necessary for proper developmental progression.

Each of the tobacco constituents identified as embryotoxic may act independently, or one or more may act in synergism to exert skeletal embryotoxicity. In turn, two chemicals identified as embryotoxic to developing skeletal cells during individual exposures may nullify embryotoxic outcomes when they act antagonistically during co-exposure. Similarly, chemicals inert with respect to skeletal embryotoxicity may exacerbate the negative outcome when exposed in combination. One such example was the addition of nicotine to catechol exposures. However, no relationship between the two is described in the literature that could explain this effect.

By itself, nicotine is a powerful neurotoxin that interacts with acetylcholine receptors and can alter brain development, though its effects are largely dependent on duration, dose, and habitual use [85,86,87]. Nicotine has been shown to have dose-dependent inhibition on cell viability and proliferation for a variety of tissues [88], though there are conflicting studies regarding the ability of nicotine to impede cell viability. However, in non-neural cells it can activate antagonistic cell survival and death pathways, though the precise mechanism of choosing one biological process over another is not clear [89]. An alternative explanation might be that nicotine needs to be metabolized to exert its toxic functions, similar to B(a)A. In line with this notion is a recent study that identified craniofacial defects in developing zebrafish upon nicotine exposure [90], a phenotype similar to that identified by us in zebrafish upon exposure to aqueous smoke extracts [18]. These facts may help to resolve the discrepancy between its contradicting cytotoxicity results and explain why cell viabilities were maintained throughout all tested concentrations in both the adult fibroblast and the immature, unspecified stem cells.

In addition to the specific catechol–nicotine interaction discussed above, toxicities of many chemicals worsened in combination with coumarin. Coumarin is a natural product and has many purposes; however, reports have indicated coumarin is not genotoxic at low doses. Yet, the compound does have seemingly dose-dependent effects on multiple different organ systems across species [91]. In human cells in vitro, IC_50_ values for hFF and H9 cells were in range of each other. This not only suggested a cytotoxic effect provoked by exposure but also was in line with toxicological studies conducted in zebrafish and Xenopus that provided half-maximal activity values for viability that were adjacent to both our own hESC and hFF IC_50_ values [65,66]. While the calculated coumarin plasma concentration reached upon consumption of 1 g of tobacco, for example, in the form of chewing tobacco, again by assuming a plasma volume of 3000 mL and founded on a measured maximum coumarin content of 1.656 µg/g of tobacco [39,40], is 19.5-fold less than our identified ID_50_ concentration, this concentration might very well become relevant when coumarin is combined with additional constituents that enhance its detrimental effect. Taken together, coumarin is likely detrimental to a variety of cell types and tissues by impacting a multitude of pathways across species.

In turn, co-exposure to benzo[a]pyrene, which was in itself categorized as a weak embryotoxicant, was beneficial to a variety of combinations. Only in combination with acetaldehyde, catechol, and coumarin did B(a)P reduce calcification below 50%, and only in the case of coumarin did it reduce cell viability. This is somewhat expected given that there is a known negative association between acetaldehyde and benzo[a]pyrene regarding hamster mortality and DNA adduct formation in human mammary epithelial cells [92,93]. In A549 adenocarcinomic human alveolar basal epithelial cells, acrolein co-exposure reduced the B(a)P-mediated increase in cellular glutathione levels [92], suggesting that these two chemicals might oppositely respond to oxidative stress. This previously noted relationship between the two chemicals provides one potential explanation for the beneficial combinatorial response, given that oxidative stress seems involved in driving the osteotoxic response to tobacco [84]. The combination of acrolein and B(a)P also reduced p53 DNA binding activity in comparison to B(a)P alone [94], greatly reducing the DNA repair capacity for damage induced by B(a)P alone, another mechanism that seems to contribute to the skeletal toxicity associated with tobacco in the hESC system [84].

Overall, whether or not chemicals elicit an embryotoxic response in differentiating skeletal cells will depend on the nature of the chemicals, their combined actions, and their actual concentrations in tobacco smoke in relation to the IC_50_ concentrations found here. Focusing on the mixture as a whole is advantageous, as it can provide simplicity of inference, integrate over multiple exposures that likely originate from similar sources, and often map directly onto the effects of potential public health interventions. These results are especially useful, as many tobacco constituents are also encountered in the agricultural and pharmaceutical sectors. Furthermore, because the parameters inserted into the ToxPI GUI were broad, they can be applied to various products that are made up of a concoction of chemicals and are not limited to those chemicals in tobacco products and smoke.

## 5. Conclusions

Exposure of differentiating human cells to chemicals found in tobacco and its products illustrated a significant effect on cytotoxicity and/or osteogenic differentiation, with catechol, coumarin, acetaldehyde, and acrolein representing chemicals that were most likely to elicit defective skeletogenesis in vivo. However, due to the altered behavior of the differentiating skeletal cells in response to combinatorial exposures, whereby toxic effects of individual chemicals were either exacerbated or cancelled within combinatorial exposures, no one single chemical could be pinpointed as the culprit of reduced calcification in response to tobacco exposure. These findings let us conclude that it may be difficult to isolate single chemicals as the primary drivers of embryotoxicity and that the full combination of chemicals in tobacco smoke may produce the hypomineralization phenotype. However, it does appear that the combinatorial relationship could be further reduced to a single constituent, provided that a more intensive look at each constituent’s mechanism of action is further evaluated.

## Data Availability

Data are contained within the article.
